# SCF, Regulated by HIF-1α, Promotes Pancreatic Ductal Adenocarcinoma Cell Progression

**DOI:** 10.1371/journal.pone.0121338

**Published:** 2015-03-23

**Authors:** Chuntao Gao, Shasha Li, Tiansuo Zhao, Jing Chen, He Ren, Huan Zhang, Xiuchao Wang, Mingxiao Lang, Jingcheng Liu, Song Gao, Xiao Zhao, Jun Sheng, Zhanna Yuan, Jihui Hao

**Affiliations:** Tianjin Medical University Cancer Institute and Hospital, National Clinical Research Center for Cancer, Key Laboratory of Cancer Prevention and Therapy, Department of Pancreatic Cancer, Tianjin, 300060, China; University of Nebraska Medical Center, UNITED STATES

## Abstract

Stem cell factor (SCF) and hypoxia-inducible factor-1α (HIF-1α) both have important functions in pancreatic ductal adenocarcinoma (PDAC). This study aims to analyze the expression and clinicopathological significance of SCF and HIF-1α in PDAC specimens and explore the molecular mechanism at PDAC cells in vitro and in vivo. We showed that the expression of SCF was significantly correlated with HIF-1α expression via Western blot, PCR, chromatin immunoprecipitation (ChIP) assay, and luciferase assay analysis. The SCF level was also correlated with lymph node metastasis and the pathological tumor node metastasis (pTNM) stage in PDAC samples. The SCF higher-expression group had significantly lower survival rates than the SCF lower-expression group (p<0.05). Hypoxia up-regulated the expression of SCF through the hypoxia-inducible factor (HIF)-1α in PDAC cells at the protein and RNA levels. When HIF-1α was knocked down by RNA interference, the SCF level decreased significantly. Additionally, ChIP and luciferase results demonstrated that HIF-1α can directly bind to the hypoxia response element (HRE) region of the SCF promoter and activate the SCF transcription under hypoxia. The results of colony formation, cell scratch, and transwell migration assay showed that SCF promoted the proliferation and invasion of PANC-1 cells under hypoxia. Furthermore, the down-regulated ability of cell proliferation and invasion following HIF-1α knockdown was rescued by adding exogenous SCF under hypoxia in vitro. Finally, when the HIF-1α expression was inhibited by digoxin, the tumor volume and the SCF level decreased, thereby proving the relationship between HIF-1α and SCF in vivo. In conclusion, SCF is an important factor for the growth of PDAC. In our experiments, we proved that SCF, a downstream gene of HIF-1α, can promote the development of PDAC under hypoxia. Thus, SCF might be a potential therapeutic target for PDAC.

## Introduction

Pancreatic ductal adenocarcinoma (PDAC) is a highly malignant tumor with poor prognosis. Understanding the molecular basis of the disease is highly desirable for developing new strategies to prevent and treat PDAC [1].

Stem cell factor (SCF), also known as a mast cell growth factor, steel factor, and kit ligand [2], is a multifunctional cytokine involved in tumor progression. SCF and its receptor, c-kit ligand (KL), are up-regulated in particular human malignancies including gastrointestinal stromal tumor (GISTs) [3], breast cancer [4,5], hematopoietic cell [6], myeloid leukaemia [7], and glioma [8]. The binding of SCF to c-kit causes receptor dimerization and protein kinase activation and mediates a variety of biological effects in tumor by many signal transduction pathways [9,10]. Recently, more and more studies showed that the SCF/c-kit system has an important function in angiogenesis, proliferation, and invasion in tumor cells [11]. Moreover, the SCF/c-kit binding has been reported to increase hypoxia-inducible factor-1α (HIF-1α) protein synthesis by the PI3K and Ras/MEK/ERK pathways in pancreatic cancer cells under normoxia, and hypoxia up-regulated SCF gene expression in breast cancer cells through HIF-1α [5]. However, the interaction between HIF-1α and SCF in pancreatic cancer remains unclear.

As an important transcription factor, HIF-1α has important functions in cancerous transformation, chemoradiotherapy resistance, and tumor progression [12]. Tumor cells increase the expression of HIF-1α by activating AKT under normoxia [13]. HIF-1α regulates many downstream genes, such as erythropoietin, VEGF, heme oxygenase-1, enolase, lactate dehydrogenase A, and aldolase [14,15]. The HIF-1α expression level was high in pancreatic cancer, and HIF-1α was related to clinical stage and lymph node metastasis [16]. Therefore, HIF-1α had been considered as a new therapeutic target for pancreatic cancer, and targeted therapy against HIF-1 expression in PDAC was recently investigated [17–19].

In the present study, we investigated the prognostic value of HIF-1α and SCF protein expression in primary PDAC tissues. The correlation between HIF-1α and SCF was explored and verified both in vitro and in vivo. Moreover, the biological effects of SCF on PDAC were investigated in vitro.

## Materials and Methods

### Cell cultures and treatments

BxPC-3 and PANC-1 cell lines were chosen for this experiment. They were obtained from the Committee of Type Culture Collection of the Chinese Academy of Sciences (Shanghai, China). PANC-1 was maintained in Dulbecco's modified Eagle's medium (Hyclone, USA) supplemented with 10% fetal bovine serum (Gibco, USA). BxPC-3 cells were maintained in RPMI-1640 medium (Hyclone, USA) supplemented with 10% fetal bovine serum (Gibco, USA). The cells were incubated at 37°C in a humidified atmosphere of 95% air and 5% CO_2_. For the hypoxia treatment, the cells were placed in a modular incubator (Thermo Electron Co, Forma, MA) consisting of 94% N_2_, 5% CO_2_ and 1% O_2_.

### Reagents and antibodies

For IHC analysis: mouse monoclonal SCF antibody (sc-13126, 1:100 dilution) and mouse monoclonal HIF-1α antibody (sc-13515, 1:100 dilution) were obtained from Santa Cruz Biotechnology.

For western blot analysis: mouse monoclonal HIF-1α antibody (sc-13515, 1:500 dilution), mouse monoclonal β-actin antibody (sc-8432, 1:2500 dilution), and rabbit polyclonal SCF antibody (sc-9132, 1:1000 dilution) were obtained from Santa Cruz Biotechnology. The secondary antibodies preparation was either anti-rabbit (1:5000) or anti-mouse (1:5000).

For cell functional experiments: rabbit polyclonal neutralizing SCF antibody (ab9716, 0.01 μg/mL) was obtained from Abcam.

### Ethics statement

The use of human samples in this study was approved by the Ethics Committee of Tianjin Cancer Hospital. The research involving human participants and animal experiments had been approved by our hospital and our equivalent committee. The participants provided their written informed consents to participate in this study, but these cannot be included in the report because of the large volume and the consents were written in Chinese.

All animal experiments were conducted according to relevant national and international guidelines.

This study was carried out in strict accordance with the recommendations in the Guide for the Care and Use of Laboratory Animals of the National Institutes of Health (Tianjin Cancer Hospital). The protocol was approved by the Committee on the Ethics of Animal Experiments of the Tianjin Cancer Hospital. All surgery was performed under sodium pentobarbital anesthesia, and all efforts were made to minimize suffering.

### Immunohistochemistry (IHC)

After obtaining the approval from the Ethics Committee, PDAC tumor samples were obtained from 95 patients (ages, 36–79 y) undergoing surgical resection, with IHC diagnosis of PDAC at the Tianjin Cancer Institute & Hospital between July 1997 and April 2010. Specimens were cut, deparaffinized, and rehydrated with xylene and graded alcohols. Antigen retrieval was carried out in 5 mM citrate buffer. After the inactivation of endogenous peroxidase with 3% H_2_O_2_, the sections were blocked with goat serum and incubated with either HIF-1α antibody (1:100) or SCF antibody (1:100) overnight at 4°C. The sections were first rinsed in phosphate buffered saline (PBS), incubated with biotinylated secondary antibody at 37°C for 20 min, and then washed with PBS three times. Diaminobenzidine was used as a chromogen substrate. Finally, the sections were counterstained with haematoxylin. The results of IHC staining were evaluated as follows: the intensity of tissue staining was graded as low shading (1), medium shading (2), and high shading (3). The proportion of positive cells was assessed as 1 (1%-33% cells stained), 2 (33%-67% cells stained), and 3 (>67% cells stained). The cases were classified into positive groups (2–3. low; 4–6. medium; >6. high) by the product of the intensity and proportion of the immunostained cancer cells for HIF-1α or SCF. Two independent pathologists evaluated the slides, and all cases with discrepant interpretations were discussed using a double-headed microscope until a consensus was reached. The clinicopathologic data and patient outcomes were not given to both pathologists.

### Western blotting

Total cell extracts were lysed using a RIPA lysis buffer (Beyotime, China) supplemented with proteinase inhibitors cocktail (Sigma, USA). The protein concentration was measured using the BCA assay kit (Sigma, USA). First, 20 μg of protein from total cell lysates was separated by 10% SDS–polyacrylamide gel electrophoresis then transferred to PVDF membranes (Invitrogen, USA). The membranes were then incubated in a blocking buffer containing 5% nonfat dry milk for 1 hr at room temperature and were incubated with the different primary antibodies overnight at 4°C. The membrane samples were then washed with TBS-T 3 times and incubated with the corresponding secondary antibodies for 1 hr at room temperature. We used ECL western blotting substrate (Pierce, USA) to test the immunoblotted bands. The primary antibody preparations were as follows: HIF-1α antibody, SCF antibody, and β-actin antibody.

### Real-time PCR analysis

Total RNA was extracted from the two pancreatic cancer cell lines by using a TRIzol reagent (Invitrogen, USA). Total RNA aliquots (1μg) were reverse transcribed with oligo (dT) primers at 70°C for 10 mins. They were then placed on ice for 2 min, and they were added to the 8U Reverse Transcriptase reaction liquid (TaKaRa, China). The mixture was then incubated at 42°C for 60 mins then at 70°C for 10 mins. Reaction mixture aliquots (cDNA) (2μl) were used as templates for RT-PCR. PCR cycling conditions were 95°C for 15 min and 40 cycles of 95°C for 15 s, 60°C for 30 s, and 72°C for 30 s, followed by the final melting curve program. β-actin RNA was used as the loading control. Each sample was done in triplicate, and the mean values were used for quantization. The primers for HIF-1α, SCF, and β-actin were as follows: forward 5'-GCAAGCCCTGAAAGCG-3' and reverse 5'-GGCTGT CCGACTTTGA-3' (HIF-1α); forward 5'-CTGCTCCTATTTAATCCTCTCGTCA-3' and reverse 5'-ATTGTACTACCATCTCGCTTATCCA-3' (SCF); forward 5'-CAGAGCAAGAGAGGCATCC-3' and reverse 5'-CTGGGGTGTTGA AGGTCTC (β-actin).

### Chromatin immunoprecipitation assay (ChIP)

A commercial chip assay kit (Upstate Biotechnology, Waltham, USA) was used following the manufacturer’s instructions. After treatment, each sample group was incubated with 1% formaldehyde to cross-link the DNA-protein complexes. The cross-links were heated at 37°C for 10 min. After being washed with cold PBS, the cells were collected and lysed with SDS lysis buffer (1% SDS, 10 mM EDTA, 50 mM Tris, pH 8.1) containing protease inhibitors. Lysate was sonicated to shear the DNA to lengths between 200 and 1000 bp. The cross-linked protein was then immunoprecipitated using mouse anti-human HIF-1α monoclonal antibody (1:100) or non-specific IgG antibody (as the negative control of the antibody, Sigma, USA). The DNA was extracted via the phenol chloroform method and precipitated by ethanol for PCR amplification. Primers flanking the hypoxia response element (HRE) of the VEGF promoter were used as the positive control. Meanwhile, primers that do not include HRE were used as a negative control. Additionally, the input and anti-RNA polymerase were used as the other positive controls of the experiment [5]. The forward and reverse primers were as follows: 5'-GCCTGCTTCTCGCCTACC-3' and 5'-GAGCTCCAGCATATTGCACG-3' (SCF), amplified the following sequence of genomic DNA: GCCTGCTTCTCGCCTACCCCGGGCTCCGGAAGGGAAGGAGGCGTGTCCGGAGCAGGCGGGCGGGAACTGTATAAAAGCGCCGGCGGCTCAGCAGCCGGGCTTCGCTCGCCGCCTCGCGCCGAGACTAGAAGCGCTGCGGGAAGCAGGGACAGTGGAGAGGGCGCTGCGCTCGGGCTACCCAATGCGTGGACTATCTGCCGCCGCTGTTCGTGCAATATGCTGGAGCTC (228bp); 5'-GCCTCTGTCTGCCCAGCTGC-3' and 5'-GTGGAGCTGAGAACGGGAAGC-3' (VEGF). The sequences of the negative primers were as follows (not including HRE): forward: 5'-CGAGACCGGCGGGAGG-3', reverse: 5’-CGGAGCCCGGGGTAGG-3'. The PCR products were separated using 1% agarose.

### Transient transfection and luciferase assay

The cells were plated at a density of 5×10^5^ cells/well in 6-well plates. The pcDNA3.1-HIF-1α plasmids were prepared as previously described. The full-length SCF luciferase promoter plasmids containing the lengths of SCF 5’-flanking sequences (spanning from +184 to-2185) were constructed and named pGL3-SCF. Mutant SCF promoter was constructed and named pGL3-SCF-M. The constructs were mutated from GCGTG to GTAGA that contained the SCF promoters but without the HRE site. We then transfected the pcDNA3.1-HIF-1α plasmids (1 μg) with either pGL3-SCF (1 μg) or pGL3-SCF-M (1 μg) into PANC-1 cells. Renilla luciferase construct (10 ng) (pRL-SV40, Promega, USA) was co-transfected as the internal reference. PGL3-Basic (Promega, USA) and pGL3-VEGF were chosen as the negative and positive control, respectively. After transfection for 48 hrs, the cells were incubated in hypoxia for 12 hrs. The luciferase activity was then determined using the Dual-Luciferase Reporter Assay System (Promega, USA). All data was normalized to Renilla luciferase expression. VEGF luciferase reporter construction (pGL3-VEGF) was used as the positive control for the HIF-1α response.

### Colony formation assay

The cells were seeded in six-well plates at a density of 200 cells per well and cultured at 37°C for two weeks. At the end of the incubation period, colonies were stained with a three-step stain set (Thermo Scientific) and counted using an optical microscope. Each measurement was performed in triplicate, and each experiment was conducted at least three times.

### Cell scratch assays

The pancreatic cells with different treatment were seeded to full confluency in 6-well plates overnight. The following day, a scratch was introduced in the middle of the well by using a sterile pipette tip. The medium was discarded and replaced with a fresh one. The rate of migration towards the center of the wound was determined after 12 hrs.

### Cell invasion assays

The invasion assays were performed with an 8.0 μm pore inserts in a 24-well Trans-well chambers (Costar, USA). For this assay, 1×10^5^ cells were isolated and added to the upper chamber of a trans-well with DMEM. The invasion assay was performed using 1/6 diluted matrigel (BD Bioscience)-coated filters. The DMEM with 10% fetal bovine serum was added to the lower chamber and incubated for 24 hours. Cells that had migrated to the bottom of the filter were stained with a three-step stain set (Thermo Scientific). Each experiment was repeated at least three times.

### Animal experiments

PANC-1 cells (1×10^6^) were injected subcutaneously into the right flank of female nude mice. Digoxin and saline for injection were obtained from Tianjin Medical University Cancer Institute and Hospital. The cells were harvested by trypsinization, washed in PBS, resuspended at a 1:1 solution of PBS: Matrigel, and injected subcutaneously into the right flank of nude nu/nu mice. When the tumors had grown to 200 mm^3^, the mice were randomized into two treatment groups (n = 8 for each group): saline control and digoxin groups. The mice received daily intraperitoneal injections of either saline or digoxin (2 mg/kg) to inhibit HIF activity [20–22]. The tumor was palpable 5 days after inoculation, and all of the mice had developed tumors by the end of the experiment. The physical condition of the animals, including fur-roughing, shedding, and local trauma at the site of injection, as well as decrements in general animal activity were regularly monitored. Tumor volume was calculated using the following formula: V = π/6 × length × width^2^. The mice were immediately euthanized after the tumor samples were isolated and photographed. All tumor samples were paraffin-embedded for IHC analysis.

### Statistical analysis

All data analyses were performed using the SPSS13.0 statistical analysis software. Differences between two samples were analyzed via unpaired t test. Multiple group comparisons were performed via χ^2^ test. Grade material related analysis was performed via Spearman's rank. Survival analysis were performed via Kaplan-Meier and log-rank test. Differences were considered statistically significant at p < 0.05.

## Results

### SCF and HIF-1α are over-expressed in PDAC and their expression levels predict poor outcome

**Fig 1:**
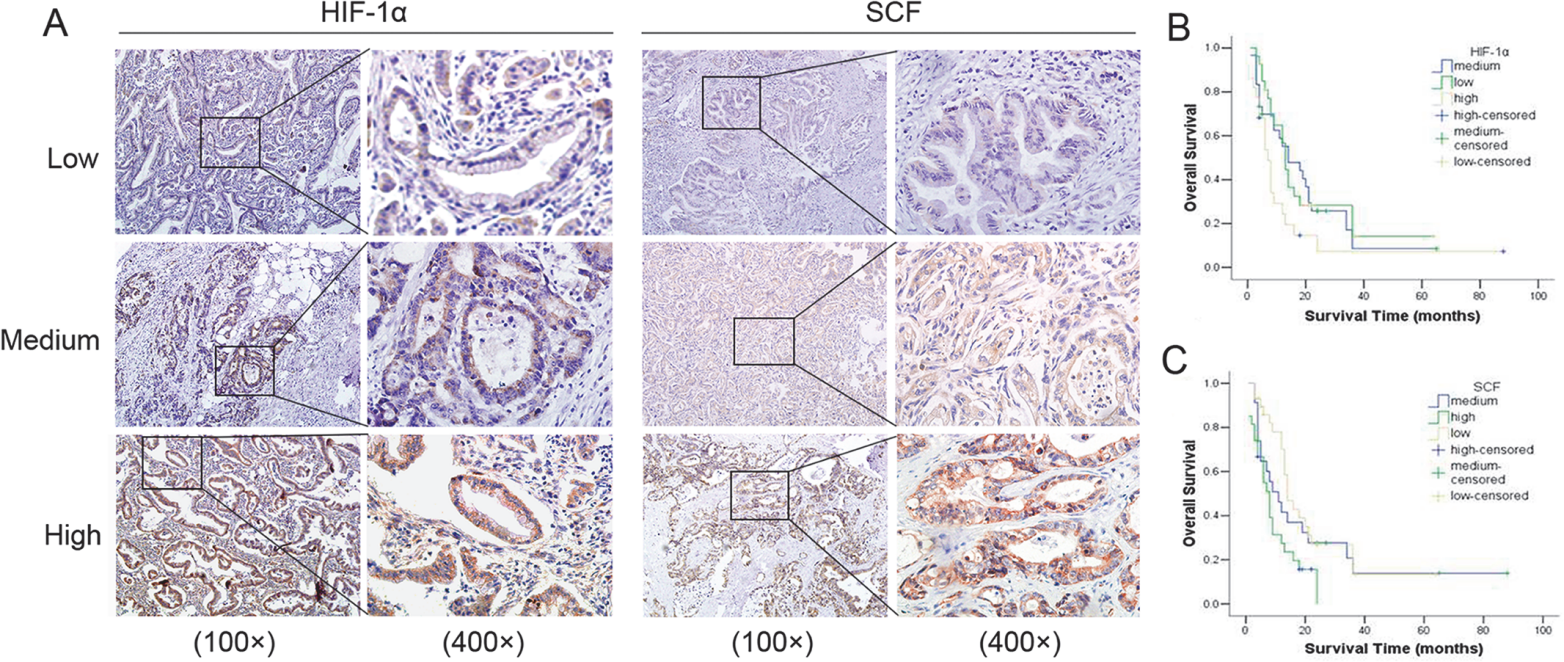
HIF-1α and SCF expression in pancreatic adenocarcinoma tissue and the cumulative survival analysis. (A): The IHC results of HIF-lα and SCF. SCF protein expression was significantly correlated with HIF-lα, as detected by immunohistochemical staining on PDAC. Left panels: various expression levels of HIF-lα protein. Right panels: expression of SCF protein of the same samples in the adjacent section. (B): The results of survival analysis on HIF-lα. PDAC patients (n = 95) with high positive HIF-lα protein expression had significantly worse total survival than those with low or medium positive expression. (C): The results of survival analysis on SCF. The same PDAC patients (n = 95) with high positive SCF protein expression had significantly worse total survival than those with low or medium positive expression.

To investigate the relationship between HIF-1α and SCF in PDAC, we examined their expression pattern in human pancreatic adenocarcinoma tissue. IHC was performed on serial sections of 95 PDAC samples. We observed that HIF-1α and SCF have higher expression in cancer samples (Fig. 1A). The Spearman analysis showed that an obvious correlation exists between SCF and HIF-1α (r = 0.728, p<0.001: S1 Table). The SCF level was correlated with the lymph node metastasis (r = 0.281, p<0.05: Table 1) and the pathological tumor node metastasis (pTNM) stages (r = 0.353, p<0.05) in these cancer samples. The results showed that HIF-1α or SCF higher-expression group had significantly lower survival rate than the HIF-1α or SCF lower-expression group (p<0.05, respectively: Fig. 1B and C).

**Table 1:** Correlations between clinicopathological features and SCF expression in PDAC.

		SCF				
		Low	Medium	High	r_s_	p-values
Age ^a^		59 (36–79)			5.747^a^	0.06
Tumor size (cm)^b^		3.649±1.67	4.435±1.359	6.107±2.99	10.809^b^	<0.001
Histological grade					0.073	0.48
G1		16	13	10		
G2		9	6	6		
G3		14	7	14		
LN metastasis					0.281	0.006
N 0		22	11	7		
N 1		17	15	23		
pTNM stage					0.353	0
I,II		23	13	6		
III		9	5	8		
IV		7	8	16		

* p-values were calculated by Spearman's Rank-Correlation test (n = 95)

Age^a^: Expressed as median (range), χ^2^ = 5.747, p = 0.060 (kruskal-wallis test)

Tumor size (cm)^b^: Expressed as mean, F = 10.809, p<0.001 (anova test)

These results indicate that SCF and HIF-1α were over-expressed in PDAC, and their expression levels can predict poor outcome.

### Expression of HIF-1α and SCF in pancreatic cancer cells under hypoxia

**Fig 2:**
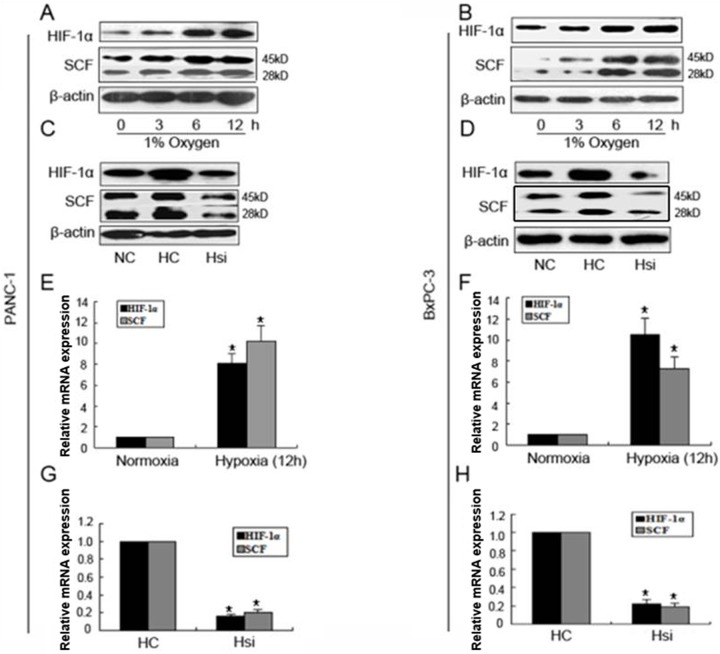
Expression of HIF-1α and SCF protein and mRNA after different treatment. (A, B): Expression of HIF-1α and SCF protein after hypoxia treatment. Two pancreatic cancer cell lines, PANC-1 and BxPC-3, were incubated under hypoxia at different time points (0, 3, 6, 12 hrs). Both of the protein expression of HIF-1α and SCF were determined via Western blot. β-actin expression was used as the control. (C, D): Expression of HIF-1α and SCF protein after transfected with siRNA targeting HIF-1α. The PANC-1 and BxPC-3 cancer cells were transfected with siRNA targeting HIF-1α for 48 hrs and incubated under hypoxia for 12 hrs. The protein expressions of HIF-1α and SCF were then determined via Western blot. (E, F, G, H): Expression of HIF-1α and SCF mRNA after different treatments. After hypoxia treatment or interference of siRNA-HIF-1α treatment in PANC-1 and BxPC-3 cancer cells, the mRNA expressions were quantified via real-time PCR. Data from three experimental determinations and bars indicate the SD, p <0.05 vs. control.

The relationship between SCF and HIF-1α was further investigated under hypoxia in vitro. The results revealed that two pancreatic cancer cell lines, PANC-1 and BxPC-3, expressed little HIF-1α and SCF protein under normoxia, and their expressions were obviously increased after hypoxia treatment, especially at 12 hrs (Fig. 2A and B). As a positive control, VEGF expression increased correspondingly (data not show). This result indicates that hypoxia up-regulated the HIF-1α and SCF protein expression in PANC-1 and BxPC-3 pancreatic cancer cell lines. To investigate whether HIF-1α regulate the expression of SCF, siRNA targeting HIF-1α was used. The two pancreatic cancer cell lines were transfected with siRNA targeting HIF-1α and cultured under hypoxia (1% O_2_) for 12 hrs (defined as Hsi, the same below), and the two cell lines only cultured under hypoxia (1% O_2_) for 12 hrs were used as the control sample (defined as HC, the same below), as shown in Fig. 2C and D. After interference in PANC-1 and BxPC-3 cell lines, HIF-1α and SCF protein levels decreased evidently compared with those of control groups. The results indicate that siRNA targeting of HIF-1α partially or completely block the expression of SCF under hypoxia. The SCF and HIF-1α mRNA expressions were then tested via quantitative RT-PCR after hypoxia treatment or siRNA targeting HIF-1α transfection culture. The results indicate that both mRNA levels of HIF-1α and SCF increased or decreased with the same trend (Fig. 2E, F, G and H): In PANC-1 cells, compared with normoxia control, HIF-1α expression was increased for 8.1 fold and SCF was increased for 10.2 fold because of hypoxia treatment, and in BxPC-3 cells, HIF-1α expression was increased for 10.5 fold and SCF was increased for 7.3 fold because of hypoxia treatment. After HIF-1α knockdown at hypoxia environment in PANC-1 cells, HIF-1α expression was reduced to 84% and SCF was reduced to 80%. In BxPC-3 cells, HIF-1α expression was reduced to 78% and SCF was reduced to 81%. These results indicate that the up-regulation or suppression of SCF expression is directly related on the regulation of HIF-lα.

### HIF-1α directly binds to the SCF promoter and up-regulated the SCF promoter activity

**Fig 3:**
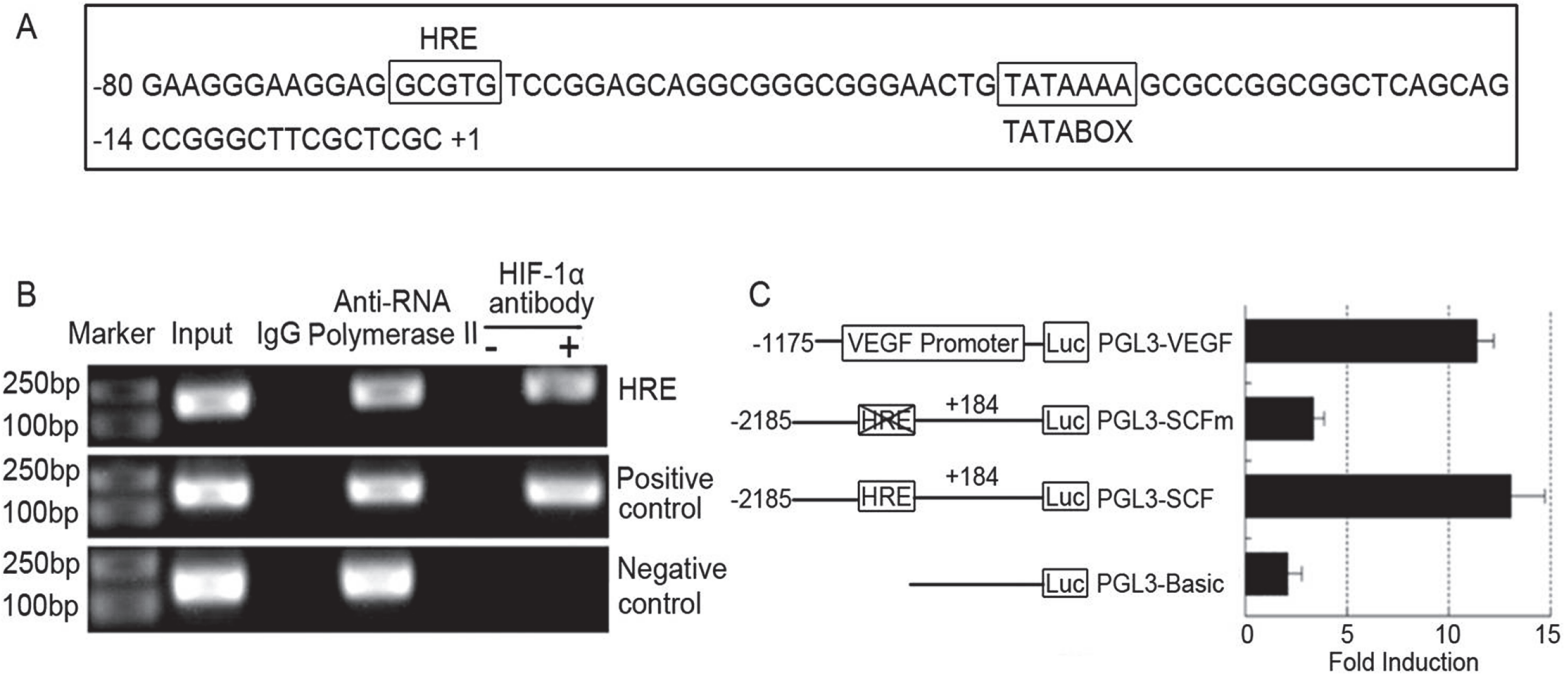
HIF-1α directly binds to the HRE region of SCF promoter and up-regulates the activity. (A): The DNA sequence of the SCF promoter. A potential HIF-1α binding site is located at −68 to −64 near the start site of transcription. (B): Chromatin immunoprecipitation analysis. PANC-1 cells were cultured under normoxia and hypoxia for 12 h and then analyzed. The PCR products of SCF promoter were only detected in the samples that were precipitated by HIF-1α antibodies but not in control IgG samples. The SCF promoter region (-1163 to-955) that do not contain HRE (non-HRE) cannot be precipitated by HIF-1α antibodies. (C): Dual luciferase results in different groups. After transfected with pcDNA3.1-HIF-1α plasmids (1 μg) with pGL3-SCF (1μg) or (pGL3-SCF-M) (1μg), pGL3-VEGF was used as the positive control. After transfection incubation for 48 h and incubated under hypoxia for 12 hrs, the cells were harvested for dual luciferase assay. The results showed that the full-length SCF promoter activity (pGL3-SCF) was increased for 12.8 fold after over expression of HIF-1α compared to that treated with plasmids alone, but the mutation of SCF promoters (pGL3-SCF-M) did not increase. Over-expression of HIF-1α was increased for 11.6 fold of the VEGF promoter activity compared to the controls.

After the screening the 5’-flanking region of the SCF gene, we found a potential HIF-1 binding site(-68 ∼-64)near the start site of transcription (Fig. 3A). To further investigate the relationship between SCF and HIF-1α in PDAC cell lines and to validate the binding of HIF-lα to the SCF promoter, an independent ChIP assay was performed after 12 hrs of hypoxia treatment. The promoter fragment was specially co-immunoprecipitated by HIF-lα antibody but not the negative controls (Fig. 3B). Furthermore, the SCF promoter region (-1163 to-955), which did not contain HRE, cannot be pulled down by the HIF-1α antibody, thereby suggesting that HIF-1α directly binds to the HRE region of the SCF promoter in vitro. To determine whether the binding of HIF-1α to the promoter can activate SCF, transient transfection and dual luciferase assay was performed to detect the SCF promoter activity after HIF-1α over-expression. The results show that the full-length SCF promoter activity (pGL3-SCF) was increased 12.8-fold after the over expression of HIF-1α compared to that treated with plasmids alone, but the mutation of SCF promoters (pGL3-SCFM) did not increase (p<0.05: Fig. 3C). Over-expression of HIF-1α was increased 11.6-fold of the VEGF promoter activity compared to the controls (p<0.05: Fig. 3C). Thus, HIF-1α directly transactivates SCF expression.

### Influence of SCF on the biological behaviors of PDAC cell lines

To investigate the influence of SCF on the biological behaviors of PDAC cell lines, exogenous SCF (150 ng/ml, the same below) or SCF neutralizing antibody (10 ng/ml, the same below) and siRNA was used to increase or decrease the expression of SCF in PANC-1 cell lines. Colony formation was used to detect cell proliferation ability and cell scratch or/and transwell chambers experiments were used to detect cell invasion.

**Fig 4:**
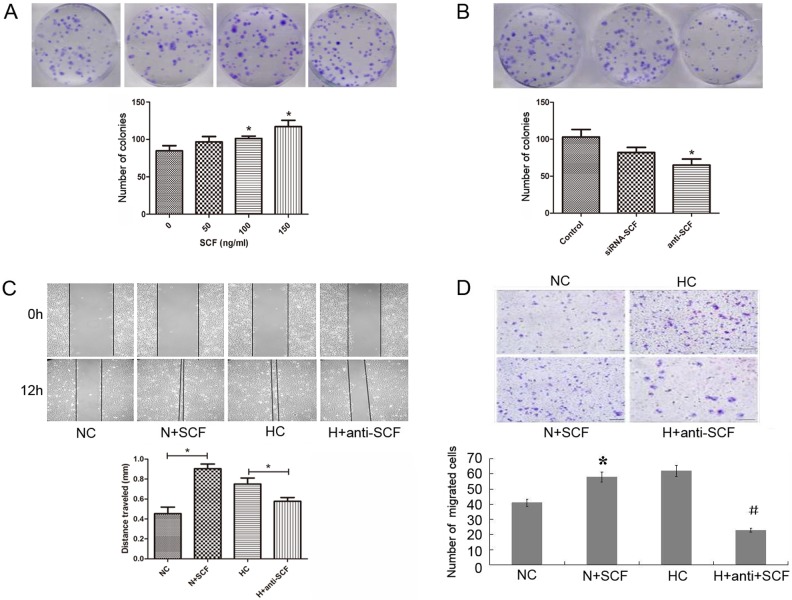
Influence of SCF on the biological behavior of PDAC cell. (A) and (B): Colony formation results showed the SCF effect on cell proliferation. After applying the interference of exogenous SCF or anti-SCF antibody in PANC-1 cells, the proliferative ability in the interference groups significantly increased or decreased compared with those of the control groups (p<0.05, respectively). * stands for p<0.05 (mark group VS control group). (C): The scratch results showed the SCF effect on cell invasion. After the interference of exogenous SCF and anti-SCF antibody, the widths in the SCF or anti-SCF groups were smaller or bigger than those in the NC or HC control group (p < 0.05, respectively). (D): The transwell chambers results showed the SCF effect on cell invasion. After the interference of exogenous SCF or anti-SCF, the cell number through the membrane was significantly more or less than the control group (p < 0.05, respectively). * stands for p<0.05 (N+SCF group VS NC group), and # stands for p<0.05 (H +anti-SCF group VS HC group).

After applying the interference of exogenous SCF and SCF neutralizing antibody or siRNA into PANC-1 cells, the proliferative ability in the interference groups significantly increased and decreased compared with those of the control groups, respectively (p<0.05, respectively: Fig. 4A and B). Similarly, after the interference of exogenous SCF and anti-SCF neutralizing antibody, the scratch space at 12 hrs of PANC-1 cells was detected. The widths in the SCF or anti-SCF groups were smaller or bigger than those in the NC or HC control group (p < 0.05, respectively: Fig. 4C). Moreover, after the interference of exogenous SCF and anti-SCF neutralizing antibody, the number of cells coming through the membrane was significantly more and less than that of the control group, respectively (p<0.05, respectively: Fig. 4D).

### HIF-1α promote the development of PDAC by regulating SCF

**Fig 5:**
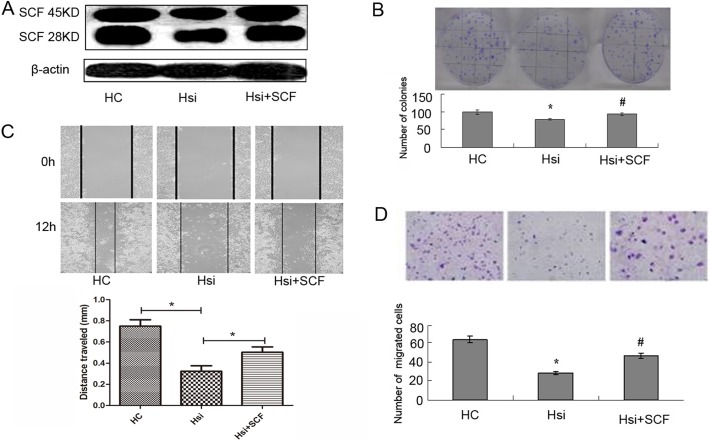
PDAC cells proliferation and invasion abilities can be rescued by up-regulation SCF following HIF-1α knockdown. (A): The levels of HIF-1α and SCF protein were tested via Western blot in different groups: The SCF protein level can be rescued by adding exogenous SCF when transfected with siRNA targeting HIF-1α at the same time. (B): Colony formation results in different groups: the decreased tumor cells proliferation rate following HIF-1α knockdown can be rescued by up-regulation of SCF (p<0.05, respectively). (C): The scratch results in different groups: the decreased tumor cells proliferation rate following HIF-1α knockdown can be rescued by up-regulation of SCF (p<0.05, respectively). (D): Transwell chambers results in different groups: the decreased tumor cells through the membrane following HIF-1α knockdown can be rescued by up-regulation of SCF (p<0.05, respectively). * stands for p<0.05 (siRNA-HIF-1α group VS control group), and ^#^ stands for p<0.05 (siRNA-HIF-1α+SCF group VS siRNA-HIF-1α group).

The results described above pointed out a possibility that HIF-1a may promote PDAC progression by regulating SCF. We rescued the cell proliferation and invasion abilities by adding exogenous SCF when transfected with siRNA targeting HIF-1α at the same time (Fig. 5A). Colony formation results showed that the decreased tumor cells growth rate following HIF-1α knockdown can be rescued by the up-regulation of SCF (p<0.05, Fig. 5B). Similarly, both cell scratch and transwell chambers results showed that the decreased tumor cell invasion rate following HIF-1α knockdown can be rescued by adding exogenous SCF (p<0.05, respectively: Fig. 5C and D).

Taken together, these results indicate that SCF, a downstream gene of HIF-1α, can promote the development of PDAC in vitro.

### Inhibiting effect of digoxin against PDAC in vivo

**Fig 6:**
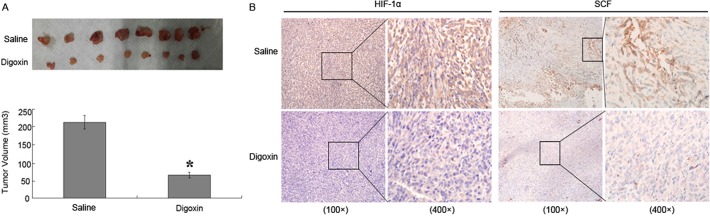
Inhibiting effect of digoxin against PDAC in vivo. (A): The mice tumors’ volume in different groups. After six weeks of treatment, digoxin therapy decreased the tumors’ volume, which was significantly smaller than those in the saline control group (p<0.05). (B): IHC results in different groups. Additionally, the IHC results confirmed the effect of digoxin: Digoxin therapy decreased the HIF-lα and SCF positive tumor cells compared to the saline control group, respectively.

To determine whether HIF-1α regulate SCF expression in vivo, we injected PANC-1 cells subcutaneously into the right flank of nude nu/nu mice. The results were as follows: compared with the saline control group, the average tumor volume in digoxin group was reduced significantly (p<0.05, Fig. 6A). Next, we evaluated the correlation between HIF-1α and SCF in the mice tumors by IHC, and the results suggest that expression of SCF was decreased as a result of the HIF-1α level reduction by digoxin (Fig. 6B). Treatment with digoxin resulted in no significant difference in the body weight of treated mice, none of the tested mice manifested signs of other adverse effects as specified in the method section, and no toxicity on the blood count or hepatic and renal function was observed with digoxin treatment (data not shown). These results indicate the anti-tumor effect and safety of digoxin in vivo.

## Discussion

In this report, we proved that HIF-1α and SCF were prognostic factors in PDAC by examining the expression of SCF and HIF-1α in pancreatic cancer tissues and analyzing the clinical features and prognosis. A strong correlation was observed between the expressions of these two factors in primary PDAC tissues. We also found that HIF-1α promoted the development of PDAC by trans-activating SCF under hypoxia in vitro. Furthermore, SCF promoted PDAC cell proliferation and invasion, and the decreased tumor cell proliferation and invasion abilities following HIF-1α knockdown can be rescued by the up-regulation of SCF under hypoxia. SCF has been suggested as a direct target gene of HIF-1 in MCF-7 cells through in vitro analysis [5]. We extended this effect in pancreatic cancers with more consolidated evidence including clinical data as well as in vitro and in vivo analysis, thereby suggesting that the regulatory role of HIF-1 on SCF is general in cancer cells. Interestingly, we recently identified SCF as an independent predictor for poor prognosis of patients with hepatocellular carcinoma [23]. In accordance with this study, SCF expression was associated with a shorter survival rate in resected patients. For those patients with high SCF expression, strengthened adjuvant therapy should be given to achieve a better outcome. Another study reported that serum analysis of PDAC also showed elevated levels of SCF in pretreated patients with pancreatic cancer [24], thereby suggesting that SCF could also be used as a diagnostic marker for pancreatic cancer. Accumulating evidences have confirmed that HIF-1 had crucial functions in the pathogenesis of pancreatic cancer. The present study further demonstrated that the proliferative and invasive effect of HIF-1 was, at least in part, mediated by SCF. Previous studies have shown that SCF activated the expression of HIF-1 in pancreatic cancer [11]. We postulate that a positive feedback loop exists between SCF and HIF. The loop may function to maintain the constitutive expression of HIF-1 under normoxia and strengthen the pathogenic effect of SCF under hypoxia.

SCF exerted its biological functions through binding to a specific ligand, c-kit. C-kit appears in embryonic tissues of human beings but was silenced in adult tissues. Recent clinical evidence showed that c-kit was negative in normal pancreatic tissues but is over-expressed in malignant pancreatic tissues, including cancerous duct, exocrine pancreas and β cells of islet, thereby suggesting that the SCF/c-kit pathway might be involved in the malignant transformation of pancreatic cells [25]. As shown in the present study, SCF mainly promoted invasion and proliferation of pancreatic cancer cells. The hypoxia-induced SCF expression might further accelerate the progression of pancreatic cancer because hypoxia is a typical microenvironment of pancreatic cancer. Another interesting function of SCF is to regulate the differentiation of PANC-1 pancreatic cancer cells into insulin-producing cells. We postulate that hypoxia might contribute to the maintenance of endocrine function of malignant pancreatic tissues through SCF/c-kit pathway.

Based on the findings of this study, we suggest that the SCF/c-kit pathway may be a potential target for the treatment of pancreatic cancer. Thus far, several c-kit inhibitors such as Imatinib and Sunitinib have been approved by FDA to the treatment of leukemia, renal cancer cell, and gastrointestinal stromal tumors [26]. Further clinical trial should be performed to use these reagents, alone or in combination with gemcitabine, as new strategies for the treatment of pancreatic cancer.

In conclusion, we identified SCF as a direct target of HIF-1 in pancreatic cancer. SCF/c-kit promoted invasion and proliferation of pancreatic cancer cells and may be a new target for the treatment of pancreatic cancer.

## Supporting Information

S1 Table Correlations between HIF-1α and SCF expression in pancreatic cancer. Statistical analysis of immunohistochemical results of HIF-1α and SCF expression in human PDAC surgical samples. p values were analyzed by Spearman’s rank-correlation test.
(DOC)
