# Supplementary material for: SCF, Regulated by HIF-1α, Promotes Pancreatic Ductal Adenocarcinoma Cell Progression
Source: PLoS One. 2015 Mar 23;10(3):e0121338. doi: 10.1371/journal.pone.0121338 (PMC4370420; doi:10.1371/journal.pone.0121338)
Supplement: S1 Table — Statistical analysis of immunohistochemical results of HIF-1α and SCF expression in human PDAC surgical samples. p values were analyzed by Spearman’s rank-correlation test. (DOC) [file pone.0121338.s001.doc]

|  | | | | | | |
| --- | --- | --- | --- | --- | --- | --- |
|  |  | HIF-1α | | |  |  |
|  |  | Low | Medium | High | r_s_ | p-values |
|  | Low | 28 | 11 | 0 | 0.728 | <0.001 |
| SCF | Medium | 4 | 21 | 1 |  |  |
|  | High | 3 | 5 | 22 |  |  |
| Spearman’s rank-correlation test n=95, r_s_=0.728 p<0.001 | | | | | | |
